# MicroRNA-216b reduces growth, migration and invasion of pancreatic ductal adenocarcinoma cells by directly targeting ρ-associated coiled-coil containing protein kinase 1

**DOI:** 10.3892/ol.2022.13363

**Published:** 2022-06-06

**Authors:** Yang-An Liu, Yue Zhang, Zhi Zheng, Kai Li, Xin-Hua Wu, Qiu-Guo Du, Xiao Ye, Lili Wang, Ling Zhu

Oncol Lett 15: 6745–6751, 2018; DOI: 10.3892/ol.2018.8109

Following the publication of this paper, it was drawn to the Editors’ attention by a concerned reader that certain of the cell migration and invasion assay data shown in Figs. 2C and 4C, and the western blot data shown in Fig. 4A were strikingly similar to data appearing in different form in other articles by different authors. Owing to the fact that the contentious data in the above article had already been published elsewhere, or were already under consideration for publication, prior to its submission to *Oncology Letters*, the Editor has decided that this paper should be retracted from the Journal. The authors were asked for an explanation to account for these concerns, but the Editorial Office did not receive a satisfactory reply. The Editor apologizes to the readership for any inconvenience caused.

